# Association of Outdoor Physical Activity and Sports with Life Satisfaction among Women of Reproductive Age According to a European Representative Sample—A Longitudinal Analysis

**DOI:** 10.3390/ejihpe13090135

**Published:** 2023-09-14

**Authors:** Viktória Prémusz, Alexandra Makai, Pongrác Ács, Evelin Derkács, Tamás Laczkó

**Affiliations:** 1 Faculty of Health Sciences, University of Pécs, 7621 Pécs, Hungary; alexandra.makai@etk.pte.hu (A.M.); pongrac.acs@etk.pte.hu (P.Á.); evelin.derkacs@etk.pte.hu (E.D.); tamas.laczko@etk.pte.hu (T.L.); 2 Physical Activity Research Group, Szentágothai Research Centre, 7624 Pécs, Hungary; 3 MTA-PTE Human Reproduction Scientific Research Group, University of Pécs, 7624 Pécs, Hungary; 4 National Laboratory on Human Reproduction, University of Pécs, 7624 Pécs, Hungary; 5 Doctoral School of Health Sciences, Faculty of Health Sciences, University of Pécs, 7621 Pécs, Hungary

**Keywords:** life satisfaction, well-being, sport, physical activity, women’s health, Eurobarometer, outdoor, reproductive age, green exercise

## Abstract

(1) Background: Low life satisfaction (LS) is associated with impaired mental and physical health. Outdoor physical activity (PA) has diverse somatic and psychological benefits. This study aimed to analyse the associations between sports settings and LS in women of reproductive age. (2) Methods: Special Eurobarometer on Sport and Physical Activity (2022, 2018, 2013) data on regularity and settings of sports/PA, LS and sociodemographic variables were analysed. The representative sample consisted of 18,489 women (34.60 ± 9.36 years). Pearson χ^2^ test and multivariate logistic regression analysis were conducted, using IBM SPSS version 28.0 according to the STROBE guidelines. The significance level was set at *p* < 0.05. (3) There was a significant difference in LS based on sports settings (χ^2^ = 409.696, *p* < 0.001). In the outdoor group, a 21.4% higher probability of being “very satisfied” compared to the non-outdoor, 30.0% higher compared to the inactive group, was found (R^2^N = 0.151). Dividing the sample by age, a significant effect remained in middle adulthood (35–44 years *p* = 0.002 and 45–49 years *p* = 0.033). (4) Conclusions: Our results underline the importance of the promotion of outdoor, green exercise and the development of special interventions to maintain or improve the psychological well-being of women in reproductive age.

## 1. Introduction

### 1.1. Background on the Benefits of Physical Activity

PA is a general umbrella term for “any bodily movement produced by skeletal muscle that results in caloric expenditure” [1] and it can be categorised in daily life into occupational, sports, conditioning, household, or other activities. Surveys differentiate PA for recreational or non-sport-related reasons and doing exercise or playing sports. Exercise has been explained for the respondents in the Eurobarometer 2022 survey as “any form of physical activity which you engage in in a sports context or sport-related setting, such as swimming, training in a fitness centre or a sports club, running in the park” [2]. Regarding the health benefits of PA, we can read about cardiovascular disease, type 2 diabetes, as well as breast, colon and reproductive cancers most often [3,4].

### 1.2. Burdens and Trends Regarding Physical Inactivity

In contrast to the above, a lack of physical activity (PA), called physical inactivity (PIA), is considered by the World Health Organization (WHO) as a critical non-communicable factor for morbidity and mortality. On the other hand, PA is taught as beneficial for the prevention of numerous diseases and conditions [5]. In the European region, PIA as a risk factor contributes to the incidence of type 2 diabetes with 12.0% and 8% of colon cancers, and 9.7% of all-cause mortality annually [6].

There are many different methods available to measure the level of PA or exercise [7,8,9]. However, it is difficult to find a larger cross-national and cross-temporal longitudinal study that was conducted using the same methodology in Europe, and the Eurobarometer survey can be mentioned as an exception. Based on Eurobarometer data of 15 European countries, PIA did not reduce in the 2013–2017 period compared to the previous surveys. Nonetheless, PIA affects more women than men. Significant gender inequity was established in 2013, with 42.1% (41.3–42.9%) of women and 35.1% (34.2–35.9%) of men (*p* < 0.001) not complying with the World Health Organization’s (WHO) aerobic PA recommendations in European Union countries, followed by 2017, where 43.8% (43.0–44.6%) of women and 39.1% (38.2–40.0%) of men (*p* < 0.001) were inactive [10,11,12]. These facts draw attention to an old need for interventions addressed to women. Promotion of the health benefits of physical activity has to be accompanied by access to enjoyable physical activity opportunities for girls and women regardless of age and socioeconomic background [4].

COVID-19 has changed our PA possibilities and habits [13,14]. PIA was examined after the COVID pandemic subsided, but still, 15% of the respondents did not walk for 10 min at a time at all weekly, and 12% were sedentary for more than 8.5 h daily. The Eurobarometer demonstrated the importance of informal settings since most PA took place in parks and outdoors (40%) at home (32%) or on the way between home and work. Based on the 2022 report, 45% of the EU population never exercise or play sport and 17% only seldom; 32% practice it with some regularity, and only 6% regularly [15]. However, to our knowledge, a detailed analysis of the Special Eurobarometer 2022 on sports regarding female participants has not yet been carried out.

### 1.3. Importance of PA in Reproductive Age

Relationships between PA and reproductive health outcomes are less well demonstrated; however, some evidence still can be found for the importance of being active for women in their reproductive age [16,17,18]. The development of evidence-based intervention advice for women in terms of promoting fertility and reproductive health is still in progress, but the relationship between the lack of PA with menstrual irregularities [19,20], ovulatory disorders such as polycystic ovary syndrome (PCOS) [21,22], longer time to conceive [23] or even infertility [24,25] and poorer response to assisted reproduction treatments [16,26,27,28,29,30,31] are known. In the case of conception, PA according to guidelines is linked with reduced risks of developing gestational diabetes, and post-partum depression [32,33,34,35]. Negative consequences were also mentioned, but an increased risk of infertility was only reported in the case of the highest levels of intensity and frequency of PA [23,36,37,38,39,40,41,42].

### 1.4. Panorama on PA and Life Satisfaction

PA is associated not only with diverse somatic advantages but also with many psychological benefits as well. It has long been proved that for women, lifelong PA promotes health and well-being throughout the lifecycle [4,43]. Increased physical activity may be beneficial among women in terms of mental health as well [44]. A positive relationship was shown between perceived autonomy support and psychological needs, which related positively to intrinsic motivation and to health goals, which in turn related positively to regular physical activity, and finally, to a positive relation with life satisfaction [45].

Life satisfaction is an integral component of an individual’s subjective well-being, which is a cognitive and evaluative assessment of an individual’s outlook on life at a given point in time [46]. Low life satisfaction was demonstrated as a predictor of depression, anxiety and neuroticism and has a reciprocal association with mental health problems [47]; furthermore, it has also been associated with impaired physical health, mortality and morbidity [48,49,50].

A higher level of overall satisfaction was reported in physically active people and improved activities of daily living (ADL), somatic and psychological health [51,52,53]. Given the level of activity, in the study of Iwon et al., active people showed a higher level of life satisfaction, compared to beginners, or inactive people and even a short engagement in a four-week physical intervention led to greater life satisfaction by beginners [54].

Self-reported medium or high physical activity level was compared to low PA as a reference category in a sample of middle-aged women. PA was associated positively with life satisfaction and negatively with depressive symptoms across all the physical performance variables. It was also revealed that PA has a stronger association with positive mental well-being than that of physical performance assessed by muscle strength, muscle power and maximal walking speed. [55].

A higher level of happiness or life satisfaction is one of the many significant benefits of doing sports. Engaging in physical activity or participating in sports is associated with improvements in life satisfaction and self-assessed health status [56,57,58].

### 1.5. Synergy of PA and Outdoor Environment

The COVID-19 pandemic has resulted in a significant change regarding the appreciation of outdoor activities. Sports venues have changed significantly during the COVID-19 epidemic. On the one hand, the restrictions affected community venues such as sports, fitness and health centres and sports clubs; on the other hand, the fear of the virus and the imperative to “stay at home” kept a significant number of people away from their usual sports venues [13,14,59,60,61].

However, the added health benefits of outdoor exercise beyond the beneficial effects of physical activity were already known before COVID-19 [51,62,63]. The WHO provided strong evidence that health and the quality of life can be improved by green infrastructure—such as parks and amenity spaces, transport corridors, gardens, green roofs, ponds, etc.—as this provides spaces for rest and restoration, physical activity, play and social interaction [64,65].

PA in natural environments is also known as green exercise (GE). This synergistic combination of exercise and exposure to nature is described by many authors highlighting a range of psychological and physiological processes. Due to evolutionary perspectives, feelings of connectedness with nature and the visual recognition of characteristic features of colours and geometrical fractals, exercising in green spaces significantly improves physiological and psychological markers of health and contributes to recovery, simply by relaxing and reducing stress exposure outside of everyday environments. By reducing tension and anxiety, improving mood and self-esteem, helping restore attention, it has benefits of activity within natural compared to more synthetic environments and improves health behaviour with fighting the growing incidence of both physical inactivity, non-communicable disease and long-term adherence to physical activity [62,66,67,68,69].

The importance of outdoor activities with special regard to PA was also demonstrated in women. Based on 10 case studies, self-esteem improved after GE, and participants with poorer self-esteem by baseline benefitted more; the main effect for gender was significant (F = 9.52, *p* < 0.01) as men had higher self-esteem than women before the intervention [63]. The effects of GE on physical health were also measured by calorie consumption per visit, which is a combination of the measurement of intensity and duration. The study revealed that significant health benefits can be implied by all intensities provided that the calories per visit increase by lower intensity all-day activities [63].

The need for social-cognitive physical interventions was shown to maximise the benefits of physical exercise among women [45]. However, to prepare an appropriate intervention, all aspects have to be investigated. The abovementioned studies indicated the existence of a relationship between sports or another PA and the settings where they engage and life satisfaction. We expect that women who exercise or practice PA outdoors regularly would show a higher level of life satisfaction than those who do not or practice non-outdoor sports.

This study aimed to examine the associations between sports settings and life satisfaction to gain a better understanding of general life satisfaction in women of reproductive age considering sociodemographic variables based on aggregated data from a multinational representative dataset of 27 European Union countries.

## 2. Materials and Methods

### 2.1. Data Sources

To regularly monitor the state of public opinion in Europe, the European Commission conducts twice a year cross-country surveys across the EU member states, with a representative sample of the population aged 15 years and more. In addition to the regular series, the polling instrument contains the Special Eurobarometer surveys, which are in-depth thematic studies [70].

The Special Eurobarometer 525, 472 and 412 on Sport and Physical Activity is a 20-question survey commissioned by the Directorate-General for Education, Youth, Sport and Culture of the European Commission, carried out by the Kantar network. Data from the last three successive rounds, with fieldwork dates of April–May 2022, December 2017 and November–December 2013 [2,11,12], were investigated. Among the topics covered, data on frequency, levels and places of engagement in sports and other physical activity were utilised.

### 2.2. Sampling

To ensure representativity, a multi-stage, random sampling method has been applied with probability proportional to population size and population density. The whole survey collected data based on face-to-face interviews in the respondents’ language from N = 26,580, N = 28,031 and N = 27,919 respondents of 27, 28 and 28 EU Member States, respectively.

From the entire sample (N = 82,530), we included women of reproductive age (N = 20,842) defined by WHO as 15 to 49 years [71], with a full record of answers regarding PA/sports questions and confounding variables (N = 18,489).

### 2.3. Measures

#### 2.3.1. Life Satisfaction

A single-item measure was used on life satisfaction from the Eurobarometer survey, “On the whole, are you very satisfied, fairly satisfied, not very satisfied or not at all satisfied with the life you lead?”. The original responses (“very satisfied”, “fairly satisfied”, “not very satisfied” and “not at all satisfied”) were utilised for the analysis.

#### 2.3.2. Physical Activity and Sports Factors

Frequency of engagement in sports and other physical activity was reported based on question QB1R (“How often do you exercise or play sport?” Regularly, With some regularity, Seldom, Never). For the respondents, “exercise” was described as “any form of physical activity which you do in a sports context or sport-related setting, such as swimming, training in a fitness centre or a sport club, running in the park”. Different settings where citizens engage in sports were analysed based on question QB10 (“Earlier you said you engage in sport or another physical activity, vigorous or not. Where do you do this?” outdoor, fitness centre, sports club, sports centre, school, at work, at home, on the way, elsewhere). Settings were defined for the respondents as places where people can practice different sports [2].

Settings were categorised as outdoor, non-outdoor and inactive. Those respondents were classified as “outdoor” who practiced sport in a park, outdoors, etc., those who did sport in any other sport-related settings were classified as “non-outdoor”, and those who indicated any other forms of PA not in a sports context or did not do any PA were classified as “inactive”. The answer “On the way between home and school, work or shops” was not considered as an outdoor activity, since it reflects active transportation in the traffic, which is presumably neither in line with the WHO’s green infrastructure concept, i.e. green transport corridors, nor a form of PE which people practice in a sports context.

#### 2.3.3. Sociodemographic Factors

The data of age categories (since the study covered women of reproductive age, original age categories 15–24, 25–34, 35–44, 45–54, 55–64, 65+ years, were modified to 15–24, 25–34, 35–44, 45–49 years), gender (only females were included), marital status ((re)married, single living with a partner, single, divorced or separated, widow, other), level of education (terminal education age, i.e., age when finished full-time education 0, 15- or less, 16–19, 20+, still studying), employment (self-employed, managers, other white collars, manual workers, house persons, unemployed, retired, students), financial difficulties (having difficulty paying bills never, from time to time, most of the time), social status (the working class of society, the lower middle class of society, the middle class of society, the upper middle class of society, the higher class of society), number of household members (one, two, three, four or more) and residence (rural area or village, small/middle town, large town) were analysed.

### 2.4. Statistical Analysis

Individuals with missing data were excluded from the analysis (N = 2353, 11.3% of total responses within the studied age and gender category). Descriptive results were presented as mean ± standard deviation (SD) absolute numbers (N) and proportion (%) with 95% confidence interval (CI).

To test categorical variables, Pearson χ^2^ test was applied. To define predicting factors of life satisfaction, a multivariate logistic regression was conducted. In the model, the dependent variable, life satisfaction, was binarily re-coded: very satisfied with the value 1, fairly satisfied, not very satisfied, and not at all satisfied with the value 0.

During the pre-testing, the predictive factors were chosen by fitting a logistic regression model using a forward selection procedure (*p* < 0.05 to enter). Eleven variables regarding socio-economic status and sporting habits available from the Eurobarometer variable set combined with the survey years were introduced for pre-testing. All variables that contributed significantly were introduced to the final prediction model and analysed according to their categories in a multivariate logistic regression analysis with the enter method.

Three goodness-of-fit assessment methods, Hosmer–Lemeshow test, Classification table and Area under Receiver Operating Characteristics (ROC) curve were applied. According to Hosmer–Lemeshow goodness-of-fit the model can be considered well-calibrated if *p* > 0.05. To estimate the discriminating power of a chosen model, the area under the ROC curve > 0.7 (with 95% confidence limits) was considered an acceptable fit. A comparison between the observed and predicted classifications was summarised by a classification table, considered a good model fit with >70% [72].

All analyses were conducted using IBM SPSS version 28.0 (SPSS Inc., Chicago, IL, USA). The significance level was set at *p* < 0.05. Considering data from a public repository, the analysis was carried out according to the STROBE guidelines if it was feasible [73].

## 3. Results

The responses of 5981 (2022), 6366 (2017) and 6142 (2013) female participants were included in the analysis with a mean age of 34.60 ± 9.36 years. Women aged 25–34 and 35–44 years were overrepresented in the sample (63.5%). Only 6.1% of them reported regular engagement in sports and 35.7% with some regularity. A high proportion, 36.3%, of the respondents never practice sports. Compared to sports, twofold of them (12.9%) take part in PA regularly and 34.2% with some regularities. The ratio of physically inactive women (never) was still high (26.1%). Regarding the type of residence, rural areas, or villages (30.3%) small/middle towns (38.8%) and large towns (30.8%) were represented balanced. Most of the respondents were married (48.7%) and lived with three or more household members together (43.5%). An average socio-economic situation was observed: participants typically terminated their studies at the age of 20+ years (38.9%), belonged to the working (27.2%), lower middle (28.2%) or middle class (34.8%) of the society and 56.0% almost never or never had fundamental financial difficulties (difficulty paying bills).

In general, it can be said that the respondents were satisfied with their lives, four out of five indicated the answer very (24.2%) or fairly satisfied (59.9%). (See Table 1) Absolute and total number confidence intervals can be found in Appendix A.

Except for residence (*p* = 0.581), significant inter-categorical difference was found in every socio-demographical variable as well as in sports (Table 1). Life satisfaction changed throughout the survey years significantly (*p* < 0.001), and the ratio of “very satisfied” respondents increased from 2013 and 2017 to 2022. In 2013 only 22.8% but in 2022 25.7% reported the highest level of life satisfaction.

The regularity of sports participation was consistent with the level of life satisfaction. Social status was somewhat inconsistent with satisfaction. Difficulties in paying bills showed that a favourable socioeconomic environment is associated with better life satisfaction. The protective effect of social relationships (being (re)married or single but living with a partner, higher number of household members) was found, although 24.3% of single women also reported they are very satisfied and 59.8% fairly satisfied with their lives. A higher level of education was in line with higher life satisfaction, but mature age (45–49 years) was related to poorer life satisfaction.

Table 2 describes the distribution of preferred sports settings among European women of reproductive age classified by survey years. The sports venues were distributed in a ratio of approximately one-third to outdoor public places, sports facilities and at work and on the way, or at home. Compared to the previous years (30.4% and 30.0%), the importance of outdoor and home activities increased to 36.1% and 35.6%, respectively. The school remains the least typical (4.3–4.8%). Besides the high increase observed in “outdoor” and “at home” activities, a significant difference was found between the data of the three years regarding, sport centre, on the way and elsewhere settings.

The outdoor subgroup had the highest proportion of respondents who were very satisfied with their lives (29.9%), and the smallest proportion of respondents who were not at all satisfied (1.6%). An inverse ratio was found in the inactive group and a lower value in the non-outdoor group compared to the outdoor (see Table 3). The Pearson χ^2^ test revealed a significant difference between life satisfaction categories based on sports settings (χ^2^ = 409.696, *p* < 0.001).

To define the predictive factors of life satisfaction, a multivariate logistic regression analysis was conducted. In the model the dependent variable, life satisfaction was binarily re-coded: very satisfied with the value 1, fairly satisfied, not very satisfied, and not at all satisfied with the value 0.

In the pre-test phase, variables regarding socio-economic status (age categories, marital status, terminal education age, employment status, financial difficulties, social status, number of household members and type of residence), sporting habits (sport settings, regularity of sport) and survey years were analysed. The eleven potential predictive factors were entered with stepwise forward selection method; all of them were selected due to their significant (*p* < 0.001) contribution to the model. The variables are summarised in Appendix B. The preliminary explanatory power of the model was 15.1% (R^2^N = 0.151).

All variables that contributed to increasing the explanatory power of the model were entered into the multivariate logistic regression model and analysed according to their categories. The reference categories for each explanatory variable are indicated in Table 4.

The final model was adjusted for sports settings and regularity of sports, social, marital and financial status, number of household members, type of residence, terminal education age (school years completed), survey years (in which year data were collected), employment and age as independent variables. The strongest predictor for life satisfaction (very satisfied) was financial status (difficulty paying bills) with a Wald value of 604.91. There was a significant difference between the categories (*p* < 0.001), it increased the probability of being very satisfied with life by 3.842 times for those respondents who almost never or never have difficulties paying the bills compared to those who had most of the time.

It is followed by the second strongest predictor, the regularity of sports participation (Wald = 116.233). Regular athletes have a 1.745 times higher odds ratio to reach high life satisfaction compared to those, who never practice sports. To practice sports with some regularity still predicted very good life satisfaction with higher probability (OR = 1.534).

Sports settings contributed also significantly to the model (<0.001). The category “Inactive” has been set as a reference and the probability of being “very satisfied” with life was 21.4% higher in women in the non-outdoor group and 30.0% higher in the outdoor group compared to inactive people. In this eleven-component model, we proved that outdoor sports have a significant effect on life satisfaction, even after introducing the well-known sociodemographic factors for which we controlled our model, outdoors still retained its significant effect.

In case of the other variables, it can be said that (re)married women, who are doing sports regularly, belong to the upper middle class, have four or more family members at home, and almost never/never have difficulties with paying bills and living in rural area or village reported with higher probability, that they are “very satisfied” with their life. Younger age was also in line with higher life satisfaction. Regarding the remaining variables (i.e., terminal education age, employment), there were no significant differences between every category, but they also contributed to the explanatory power of the model. Only in the case of the employment variable, due to redundancies, the degree of freedom has been reduced, and students were dropped from the model.

Low Nagelkerke R Square (R^2^N = 0.163) indicated a weak relationship (value of 0.2 or less) between the predictors and the outcome. To have an explanatory power of the final model by 16% is considered acceptable in the field of social sciences.

Three goodness-of-fit assessment methods, Hosmer–Lemeshow test, Classification table and Area under Receiver Operating Characteristics (ROC) curve were applied. According to the Hosmer–Lemeshow goodness-of-fit *p*-value of 0.364 (*p* > 0.05), the model can be considered well-calibrated. The model has a good diagnostics ability with a mean area under the ROC curve of 0.723 (0.714–0.731, *p* < 0.001). 76.7% (>70%) of the subjects are correctly classified by the model. All three methods of model fit assessment indicated good model fit.

We were also interested in whether age has an additional differentiating role. Dividing the sample by age, a significant effect remained in middle adulthood. In Table 5 the association of sports settings with life satisfaction was presented among women of reproductive age divided by age categories. A significant effect has only remained in the age category of 35–44 (*p* = 0.002 **) and 45–49 years (*p* = 0.033 *).

## 4. Discussion

Life satisfaction is a cognitive evaluation of a person’s general quality of life, based on the person’s criteria and subjective assessment, and is one of the important components of subjective well-being [74]. Its examination is of particular importance, since it is associated with mental and physical health, furthermore with mortality and morbidity [48,75].

Previous studies proved the benefits of health-sport on life satisfaction [54,76,77] and reproductive health [16,17,18,19,20,21,22,23], and the advantages of outdoor exercise [51,62,63] are also known. However, a multi-aspect analysis has so far been lacking. The current study aimed to examine the associations between sports settings and life satisfaction and to gain a better understanding of general life satisfaction in women of reproductive age considering sociodemographic and economic variables based on three-year multinational data from the Eurobarometer public opinion survey. According to the main hypothesis of the study, better life satisfaction was expected by women, who engaged in green exercise, i.e., sports in outdoor settings, than non-outdoor or do not engage in any sports at all.

Analysing the aggregated data of 18,489 women of reproductive age from the public dataset of the Special Eurobarometer 2022, 2018 and 2013, we found that 36.3% of respondent never practice sports, and 26.1% of them is still physically inactive (never practice PA). This is a similar tendency, but still a lower inactivity proportion compared to the Reports of the Eurobarometer surveys, which already warned, that in the European Union, the number of PIA people who never exercise or play sports continuously increases. Compared to 2013, the proportion of those who never exercise or play sports has increased from 42% to 46% in 2018 and remained at 45% in 2022, while the proportion that does so seldom has decreased (from 17% to 14%). However, still, around 60% of the population is almost inactive [2].

According to the newest Special Eurobarometer 2022 on Sport and Physical Activity, most PA takes place in informal settings, outdoors (47%), at home (37%) or on the way home or work, school, etc. (24%). Compared to the previous data collections the importance of parks and outdoors (40% in 2018, 40% in 2013) and at home (32% and 28%, respectively) increased after the COVID-19 era, but the preference to be active on the way home (23% and 21%) remain unchanged compared to 2018 [2,11,12].

Regarding women of reproductive age, we found similar tendencies, outdoors and at home were the most preferred venues and their importance increased from 30.8% to 36.1% and 33.4% to 35.6%. However, sports clubs and sports centres have lost some of their importance, by the middle data collection, the importance of sports centres has increased by roughly the same amount that training at home has decreased. Thus, a process of institutionalization began, but this was interrupted by the pandemic.

The main interest of our study was life satisfaction. In general, it can be said that the respondents were satisfied with their lives, four out of five indicated the answer very (24.2%) or fairly satisfied (59.9%). Life satisfaction varied significantly between the study years (*p* < 0.001 **). The ratio of “very satisfied” respondents has been increasing continuously throughout the survey years. In 2013, only 22.8%, but in 2022, 25.7% reported the highest level of life satisfaction. At first reading, it may be surprising given the data collection has been conducted after the calm down of COVID-19, although there were already studies by the third wave describing a much better mental state in terms of adaptation to the pandemic situation compared to the beginning of the pandemic [59,78].

Laczkó et al. reported in their longitudinal study after a dramatic decrease, an ascending level of psychological well-being (1st wave 8.57 ± 3.14, 2nd wave 8.72 ± 2.47 *p* < 0.001, 3rd wave 9.09 ± 3.11 *p* < 0.001) using the WHO-5 Well-Being Index on a national representative sample (N = 3600) during the first three waves of COVID-19. They also highlighted the protective role of sports on mental health, since they reported a positive relationship between maintaining sports or even increasing the time spent with sports and well-being during every wave of the pandemic applying OLS regression models (R^2^ = 0.233 F = 46.257 Sig < 0.001, R^2^ = 0.298 F = 63.974 Sig < 0.001, R^2^ = 0.312 F = 68.636 Sig < 0.001, respectively) adjusted for socio-demographic variables, such as age, gender, size of the settlement, education, change in income during the pandemic, physical health, and the number of the household members [78].

Rajani et al. studied the association of life satisfaction with environmental and sociodemographic factors (N =268,696) in 27 European countries based on the Standard Eurobarometer 2014–2015. They investigated both genders and found women’s level of satisfaction was more likely to be higher (OR = 0.91; 95% CI: 0.89–0.92). Just as the life satisfaction of adolescents and young adults (15–24 years old, OR = 1.79; 95% CI: 1.71–1.89) was better compared to older respondents, so was that of married respondents compared to single (OR = 0.66), widowed (OR = 0.60) or divorced respondents (OR = 0.58). Consistent with the literature, our results also showed statistically significant differences (*p* < 0.05) in life satisfaction regarding sociodemographic factors such as marital status, level of education, employment, micro- and macroeconomic status, and household size, except for residence type (*p* = 0.581). We also experienced the protective effect of social relationships, as women who were married or living with a partner scored better, but, in contrast to the previous literature, single women were similarly satisfied with their lives: 24.3% reported feeling very and 59.8% fairly satisfied.

Age was inversely related in our sample, as women in the youngest age category scored the best, although we did not examine 49+ participants [79]. The reason for this was that the present analysis is a preliminary study intended as a basis for an intervention to be developed for women of reproductive age.

Another cornerstone of the study was the regularity of sports participation and preferred sports settings. The regularity of sports participation was consistent with the level of life satisfaction. Both “regularly” and “with some regularity” similarly resulted in better life satisfaction, 32.8% and 53.3% of regular athletes were, very satisfied and fairly satisfied, while 32.0% and 59.0% of somewhat regular were very or fairly satisfied. This association was confirmed by a multivariate logistic regression model (*p* < 0.001 **, ExpB = 0.802).

Exercise or playing sports regularly was considered beneficial for life satisfaction according to Polish authors. Iwon et al. conducted a short (4 weeks) PA intervention and invited active people, beginners, and inactive people (N = 217, 124 women) to the programme to improve their subjective well-being, measured with subscales on happiness, satisfaction with life and self-esteem. Active people reported significantly higher levels of happiness and self-esteem compared to beginners and inactive people (*p* < 0.001) and a higher level of life satisfaction than inactive people (*p* < 0.05). They also proved that even a short engagement in exercise may contribute to an increase in subjective well-being. Beginners reported greater life satisfaction and happiness compared to the baseline (*p* < 0.05) [54].

The “Active and Happy” assumption could not be proven directly only with the examination of the frequency of sports participation. Satisfaction with life of 328 young, educated women (18–30 years) was assessed using the Satisfaction with Life Scale (SWLS) in Poland. Planned physical activity (frequency, duration of sessions, satisfaction with frequency, motivation and self-assessment of physical fitness was also examined. Although, physical activity does not differentiate (*b* = 0.01, *p* = 0.87) the level of life satisfaction. Multivariate analyses only confirmed the significant effect of marital status (*b* = 0.35, *p* = 0.04) and subjective assessment of physical condition (*b* = 0.29, *p* = 0.03) on life satisfaction [76].

Maher et al. also considered PA as a valuable tool for enhancing life satisfaction as well. However, according to them, to consider the level of activity is not sufficient. Their hypothesis, that there are differences across the adult lifespan. In older adults’ usual levels of PA (differences between activity level of people—between-person association) in younger adults (day-to-day differences in PA—within-person association) are more significant. Life satisfaction was measured with a single item from the Satisfaction with Life Scale and PA with the Godin Leisure Time Exercise Questionnaire. In a daily diary study with a lifespan sample (18–89 years) of 150 community-dwelling adults, they found that higher levels of both between- and within-persons PA were associated with higher levels of life satisfaction, (γ01 = 2.34, *p* < 0.05, γ10 = 0.91, *p* < 0.05, respectively). A positive association between usual (between-person) PA and life satisfaction in middle-aged and older adults was found (age difference γ05 = 0.12, *p* < 0.05). On those days when people were more active, experienced greater life satisfaction, but (within-person) association did not differ across ages (γ12 = −0.01, *p* = 0.97). Life satisfaction in general showed an inverted U-shaped distribution, it was lower by young, high by midlife and again lower by older adults [77] (Maher et al., 2015).

However, other authors were not able to show a precise dose–response of women’s health-related quality of life (HRQoL) and life satisfaction to PA. Eime et al. examined the dose–response association of health-related quality of life, life satisfaction and the self-report on duration and type of recreational PA of 793 rural-living women. According to the type of PA, they were 33.9 ± 13.7 years old in the sports club (tennis or netball) group, the 38.5 ± 12.9 years old group visited a gymnasium (resistance training or exercise classes) and the group who took leisure-time walking (alone, with up to 3 participants, or with a dog) was 44.5 ± 13.2 years old. To measure the dose–response, they categorised the PA of the respondents during the previous 7 days in tertiles, 0–150 min, > 150–350 min, and > 350 min accordingly. Walkers were over-represented in the first tertile, and sports club participants in the third tertile. Dose response to PA exposure correlated only with physical health (*p* = 0.003). Life satisfaction (*p* < 0.001) and mental health (*p* = 0.005) were associated with the type of PA.

Based on the above, the influencing factor is the type of sport rather than the regularity. This assumption was partially confirmed by Scandinavian authors but about the chosen settings for sports. It is well known that the Scandinavian countries are at the forefront of the examination of well-being and life satisfaction. Satisfaction with life as a whole and with 10 specific domains of life was investigated (LiSat-11) in an 18–64-year-old, nationally representative Swedish sample of 1207 women and 1326 men concerning education, employment situation, health and physical activity. The most prominent positive predictors were according to the logistic regression, perceived good health (OR = 3.0 (2.2–4.0)) and brought up in Sweden 2.0 (1.3–3.0). 68.9% of the study sample was active in sports/exercise and 63.3% did it outdoors. Respondents who were active in sports/exercise reported) compared to non-actives had higher levels of satisfaction with life as a whole (overall satisfaction *p* ≤ 0.001, Z = −4.6) and scored higher in health-relate (activities of daily living *p* ≤ 0.001, Z = −5.7, somatic health *p* ≤ 0.001, Z = −7.8 and psychological health 0 *p* ≤ 0.001, Z = −4.0) spare time (leisure *p* ≤ 0.001, Z = −10.3, contacts *p* ≤ 0.001, Z = −4.5) and economy items (*p* ≤ 0.001, Z = −4.8). However, being active outdoors was a positive predictor only of satisfaction with leisure (*p* ≤ 0.001, Z = −5.1). Gender was not a significant predictor of satisfaction with life as a whole [51].

In our analysis, women in the outdoor group scored higher in life satisfaction and had a 21.4% higher probability of being “very satisfied” compared to the non-outdoor group and 30,0% to inactive people. If we adjusted the model for socio-demographic and sports participation variables, the outdoors still retained 16% explanatory power. If we divided the sample by age, we realised that a significant effect remained by the two highest age categories, 35–44 (*p* = 0.002 **) and 45–49 years (*p* = 0.033 *). If we consider these facts in the reproductive health context, it underlines the importance of the promotion of green exercise interventions with special regard to late primipara and women in the age of the transition into menopause to maintain or improve their life satisfaction and thereby their psychological well-being.

The full report of Eurobarometer 2022 also highlighted that compared to the previous surveys, the two most popular sport and PA settings are still parks, outdoors, etc. and at home, and these settings have gained 7% and 5%, respectively, since 2017 regarding the full survey sample, which they consider as a consequence of the COVID-19 pandemic [15]. In our study population, we realised a similar increase in the outdoors (+5.7%), but a higher preference for home exercise existed only compared to 2017, as it only returned to 2013 levels after a 2017 decline.

Nevertheless, the authors welcome the above changes and hope that these changes will be sustainable and can be maintained after the stillstand of the COVID-19 pandemic. On the one hand, more beneficial effects were found for life satisfaction regarding green exercise compared to other sports habits, but on the other hand, these benefits may be available to people with financial difficulties. This advantage was already mentioned in the Special Eurobarometer 2022 Report. We agree that “free” settings such as parks could be a good opportunity to mobilise those respondents, for whom membership in formal sports facilities is difficult to afford. Free places could increase their engagement in sports. Unfortunately, our present results do not confirm this. In the aggregate sample (N = 18,489), respondents with less favourable socio-economic status were overrepresented in the inactive group (working class 42,9%, difficulties with paying the bills most of the time 43,7%) and underrepresented in outdoor sports (working class 27.2%, difficulties with paying the bills most of the time 25.1%). Lacking motivation or information to use the freely available facilities can be the result of lower health- and physical literacy, which is usually in line with less favourable socio-economic status [27,80,81,82] and needs to be improved with educational programs.

Instead of the preference for outdoors, according to the Eurobarometer, 15% of EU citizens did not walk for 10 min at a time at all weekly in 2022, the same as in 2017 (15%), and +2% compared to 2013. The sedentary behaviour is also still high, as 12% were sitting for more than 8.5 daily. Age progress is also present, as the desire to be active decreases, which can be seen even during walking time. The promotion of green exercise may be a good alternative since regular exercisers prefer the outdoors, and the literature also mentions the importance of outdoor places to maintain compliance for PA and sports [62]. Utilising public places for sports may be associated with the frequency of sports activity.

Understanding the correlates and determinants in women of reproductive age can provide further insights into life satisfaction measures that can be used in women’s health research. However, further examinations of their reproductive health and fertility awareness are needed in the context of life satisfaction and sport settings and participation.

### Limitations

The present study was particularly interested in women of reproductive age; it should be noted that country, regional and gender differences were not taken into account, and, in this regard, the results cannot be generalised. The sample consisted of data from 15-year-old adolescents and adults combined, even though physical activity recommendations for them differ, but the aim of the study was not to determine the sufficient level. Physical activity was only examined based on regularity and location, and the results were corrected for age categories and since the goal was to examine the reproductive age, it was necessary to integrate them based on the WHO definition. Any dataset based on self-reports can contain fundamental biases.

However, this can be considered the strength of the study, in addition to the fact that the analysis has a large, representative sample based on continuous data collection and contains high-quality, reliable time series data from Eurobarometer.

## 5. Conclusions

In the current analysis, the association of outdoor physical activity and sports with life satisfaction among women of reproductive age was investigated in a European representative sample based on longitudinal and aggregated Eurobarometer data. The results revealed a significant difference in life satisfaction based both on regularity and settings of exercise besides the socio-demographic variables. In the outdoor group, a higher probability of being “very satisfied” compared to the non-outdoor group and even higher compared to the inactive group was found. Dividing the sample by age, a significant effect remained in middle adulthood. If we consider the results in the female reproductive health context, these underline the importance of the promotion of green exercise and the development of special interventions to maintain or improve life satisfaction and thereby the psychological well-being of women in reproductive age.

## Figures and Tables

**Table 1:** Life satisfaction among women of reproductive age (15–49 years) based on a European representative sample from Eurobarometer 2022, 2018 and 2013 (N = 18,489) classified by sociodemographic and sports variables.

		Life Satisfaction						Life Satisfaction			
Variables	Categories	Very Satisfied	Fairly Satisfied	Not Very Satisfied	Not at All Satisfied	Variables	Categories	Very Satisfied	Fairly Satisfied	Not Very Satisfied	Not at All Satisfied
Survey years ^#^ *p* < 0.001 **	2022	25.7%	61.6%	10.8%	1.8%	Marital status *p* < 0.001	(Re)Married	25.2%	60.4%	12.0%	2.4%
	2017	24.3%	62.6%	11.4%	1.7%		Single living with a partner	25.3%	60.0%	12.5%	2.2%
	2013	22.8%	55.8%	16.8%	4.5%		Single	24.3%	59.8%	13.1%	2.9%
Regularity of sports *p* < 0.001 **	Regularly	32.8%	53.3%	11.9%	2.0%		Divorced or separated	13.1%	56.1%	24.3%	6.5%
	With some regularity	32.0%	59.0%	7.8%	1.1%		Widow	10.3%	55.5%	26.5%	7.7%
	Seldom	22.0%	64.0%	12.2%	1.8%		Other	32.0%	57.5%	7.8%	2.7%
	Never	16.4%	59.3%	19.2%	5.1%	Household members *p* < 0.001	One	21.1%	59.6%	15.7%	3.7%
Social status *p* < 0.001 **	Working class	15.8%	55.0%	23.3%	5.9%		Two	22.5%	59.8%	14.3%	3.5%
	Lower middle class	23.7%	62.0%	12.4%	1.9%		Three	22.7%	61.1%	13.6%	2.6%
	Middle class	27.5%	64.3%	7.1%	1.1%		Four or more	26.6%	59.4%	11.7%	2.3%
	Upper middle class	47.0%	48.3%	3.8%	0.8%	Residence *p* = 0.581	Rural area or village	24.6%	59.5%	13.1%	2.8%
	Higher class	33.5%	46.3%	14.5%	5.8%		Small/middle town	24.0%	60.3%	13.2%	2.5%
Difficulty paying bills *p* < 0.001 **	Most of the time	8.5%	43.4%	34.9%	13.2%		Large town	24.1%	59.6%	13.3%	3.0%
	From time to time	13.6%	64.9%	18.9%	2.6%	Terminal education age (years) *p* < 0.001	0	15.2%	67.4%	17.4%	0.0%
	Almost never/never	33.5%	60.5%	5.3%	0.7%		15- or less	11.7%	52.6%	27.6%	8.1%
Employment *p* < 0.001 **	Self-employed	27.5%	59.4%	10.3%	2.8%		16-19	17.7%	62.3%	16.5%	3.5%
	Managers	34.2%	58.8%	6.2%	0.8%		20+	29.9%	59.4%	9.0%	1.7%
	Other white collars	21.1%	67.1%	10.2%	1.7%		Still Studying	34.1%	57.7%	7.0%	1.2%
	Manual workers	19.5%	62.2%	15.6%	2.6%	Age categories *p* < 0.001	15-24	30.2%	57.4%	10.7%	1.8%
	House persons	24.5%	56.0%	16.1%	3.4%		25-34	25.5%	60.1%	11.9%	2.5%
	Unemployed	13.3%	49.3%	27.9%	9.4%		35-44	22.2%	60.7%	14.2%	2.8%
	Retired	11.8%	50.3%	28.8%	9.2%		45-49	19.9%	60.2%	15.6%	4.2%
	Students	34.1%	57.0%	7.7%	1.1%	Total		24.2%	59.9%	13.2%	2.8%

^#^ Pearson χ^2^, ** *p* ≤ 0.01.

**Table 2:** Distribution of sport settings among women of reproductive age in a European representative sample—aggregated data from Eurobarometer 2022, 2018 and 2013 (N = 18,489), classified by survey years.

		Outdoors	Fitness Centre	Sport Club	Sport Centre	School	At Work	At Home	On the Way	Elsewhere
**Total**	**%**	**32.3**	**13.9**	**7.3**	**6.4**	**4.6**	**10.2**	**32.9**	**23.5**	**2.2**
	N	5972	2564	1352	1180	846	1886	6086	4352	411
	CI	31.1–33.5	12.6–15.2	5.9–8.7	5.0–7.8	3.2–6.0	8.8–11.6	31.7–34.1	22.2–24.7	0.8–3.6
2022	**%**	**36.1**	**14.0**	**6.7**	**6.0**	**4.5**	**10.0**	**35.6**	**24.6**	**0.7**
	N	2036	791	377	340	256	564	2006	1385	39
	CI	34.0–38.2	11.6–16.4	4.2–9.2	3.5–8.5	1.7–7.0	7.5–12.5	33.5–37.7	22.3–26.9	−1.9–3.3
2017	**%**	**30.4**	**13.6**	**7.4**	**7.3**	**4.3**	**10.5**	**30.0**	**20.8**	**3.1**
	N	1878	842	456	452	267	648	1850	1286	191
	CI	28.3–32.5	11.3–15.9	5.0–9.8	4.9–9.7	1.9–6.7	8.1–12.9	27.9–32.1	18.6–23.0	0.6–5.6
2013	**%**	**30.8**	**13.9**	**7.8**	**5.8**	**4.8**	**10.1**	**33.4**	**25.2**	**2.7**
	N	2058	931	519	388	323	675	2230	1681	180
	CI	28.8–32.8	11.7–16.1	5.5–10.1	3.5–8.1	2.5–7.1	7.8–12.4	31.4–35.4	23.1–27.3	0.3–5.1
Sig. ^#^		*p* < 0.001 **	0.731	0.058	0.001 **	0.415	0.513	*p* < 0.001 **	*p* < 0.001 **	*p* < 0.001 **

^#^ Pearson χ^2^, ** *p* ≤ 0.01, CI: 95% confidence interval.

**Table 3:** Life satisfaction among women of reproductive age in a European representative sample—aggregated data from Eurobarometer 2022, 2018 and 2013 (N = 18,489), classified by sports settings.

			Life Satisfaction				
			Very Satisfied	Fairly Satisfied	Not Very Satisfied	Not at All Satisfied	Total
**Sport settings**	Inactive	%	18.9	60.4	16.3	4.4	34.3
		N	1200	3826	1033	280	6339
		CI	16.7–21.1	58.9–61.9	14.0–18.6	2.0–6.8	33.1–35.
	Non-outdoor	%	24.1	60.1	13.6	2.3	33.4
		N	1485	3711	839	140	6175
		CI	21.9–26.3	58.5–61.7	11.3–15.9	−0.2–4.8	32.2–34.6
	Outdoor	%	29.9	59.1	9.4	1.6	29.9
		N	1789	3530	563	94	1789
		CI	27.8–32.0	57.5–60.7	7.0–11.8	−0.9–4.1	28.7–30.1

Pearson χ^2^ = 409.696, *p* < 0.001, CI: 95% confidence interval.

**Table 4:** Association of sport and sociodemographic variables with life satisfaction among women of reproductive age in a European representative sample—aggregated data from Eurobarometer 2022, 2018 and 2013 (N = 18,489), derived from multivariate logistic regression analysis (enter method).

Variables	Categories	B	S.E.	Wald	df	Sig.	Exp(B)
Sport settings	**Outdoor** ^a^			**32.875**	**2**	**<0.001** **	
	Inactive	−0.263	0.051	26.763	1	<0.001 **	0.769
	Non-outdoor	−0.194	0.044	19.733	1	<0.001 **	0.824
Regularity of sports	**Never** ^a^			**116.233**	**3**	**<0.001** **	
	Regularly	0.557	0.080	48.403	1	<0.001 **	1.745
	With some regularity	0.428	0.051	71.069	1	<0.001 **	1.534
	Seldom	0.059	0.058	1.056	1	0.304	1.061
Social status	**Working class** ^a^			**116.232**	**4**	**<0.001** **	
	Lower middle class	0.083	0.056	2.184	1	0.139	1.086
	Middle class	0.312	0.059	28.067	1	<0.001 **	1.366
	Upper middle class	0.877	0.086	104.525	1	<0.001 **	2.403
	Higher class	0.598	0.169	12.503	1	<0.001 **	1.819
Difficulty paying bills	**Most of the time** ^a^			**604.91**	**2**	**<0.001** **	
	From time to time	0.38	0.09	17.941	1	<0.001 **	1.462
	Almost never/never	1.346	0.085	251.364	1	<0.001 **	3.842
Household members	Four or more ^a^			22.433	3	<.001	
	**One**	−0.145	0.078	3.449	1	0.063	0.865
	Two	−0.18	0.052	11.771	1	<0.001	0.836
	Three	−0.197	0.046	18.067	1	<0.001	0.821
Residence	**Large town** ^a^			**13.364**	**2**	**0.001**	
	Rural area or village	0.176	0.048	13.364	1	<0.001 **	1.192
	Small/middle town	0.089	0.045	3.908	1	0.048	1.093
Terminal education age (years)	**Still studying** ^a^			**63.206**	**4**	**<0.001**	
	0	−0.144	0.442	0.106	1	0.745	0.866
	15- or less	−0.489	0.145	11.307	1	<.001	0.613
	16–19	−0.261	0.108	5.796	1	0.016	0.771
	20+	0.052	0.108	0.234	1	0.629	1.053
Marital status	**(Re)Married** ^a^			**51.311**	**5**	**<0.001** **	
	Single living with a partner	−0.145	0.054	7.124	1	0.008	0.865
	Single	−0.361	0.062	34.245	1	<0.001 **	0.697
	Divorced or separated	−0.434	0.093	21.578	1	<0.001 **	0.648
	Widow	−0.595	0.279	4.542	1	0.033	0.551
	Other	−0.462	0.214	4.675	1	0.031	0.630
Survey years	**2022** ^a^			**16.236**	**2**	**<0.001** **	
	2017	−0.015	0.045	0.108	1	0.743	0.985
	2013	0.205	0.058	12.547	1	<0.001 **	1.228
Employment	**Self-employed** ^a^			**78.838**	**6**	**<0.001** **	
	Managers	0.084	0.079	1.135	1	0.287	1.087
	Other white collars	−0.255	0.078	10.613	1	0.001	0.775
	Manual workers	−0.107	0.078	1.876	1	0.171	0.899
	House persons	0.158	0.089	3.129	1	0.077	1.171
	Unemployed	−0.438	0.101	18.688	1	<0.001 **	0.645
	Retired	−0.443	0.202	4.813	1	0.028	0.642
Age	**45–49** ^a^			**62.313**	**3**	**<0.001** **	
	15–24	0.537	0.084	41.099	1	<0.001 **	1.710
	25–34	0.345	0.058	35.903	1	<0.001 **	1.412
	35–4	0.1	0.055	3.367	1	0.067	1.105
Constant		−2.369	0.143	274.073	1	<0.001	0.094

^a^ Reference categories. ** *p* < 0.001.

**Table 5:** Association of sport settings with life satisfaction among women of reproductive age in a European representative sample—aggregated data from Eurobarometer 2022, 2018 and 2013 (N = 18,489) divided by age categories.

	Outdoor ^a^	Non-Outdoor		Inactive		Goodness-of-Fit			
Age (Years)	Sig.	Sig.	Exp(B)	Sig.	Exp(B)	AR	HL	AUC ROC	R^2^N
15–24	0.079	0.039 *	0.784	0.077	0.827	71.80%	0.977	0.69	0.163
25–34	0.073	0.035 *	0.834	0.083	0.876	75.30%	0.529	0.711	0.151
35–44	0.002 **	0.002 **	0.771	0.005 **	0.813	78.10%	0.615	0.732	0.172
45–49	0.033 *	0.034 *	0.779	0.022 *	0.783	80.50%	0.668	0.768	0.224
Total		*p* < 0.001 **	0.797	*p* < 0.001 **	0.833				

AUC ROC: Area under Receiver Operating Characteristics (*p*-value), HL: Hosmer–Lemeshow test (*p*-value), OA: overall accuracy rate, R^2^N: Nagelkerke R Square. ^a^ Reference category: outdoor. * *p* ≤ 0.05, ** *p* ≤ 0.01.

## Data Availability

The raw datasets are owned by the European Commission and available online. **Special Eurobarometer 525:** Sport and physical activity, 2022. **Landing Page:** http://data.europa.eu/euodp/en/data/dataset/S2668_97_3_SP525_ENG (accessed on 15 June 2023). **Special Eurobarometer 472:** Sport and physical activity, 2018. **Landing Page:** http://data.europa.eu/euodp/en/data/dataset/S2164_88_4_472_ENG (accessed on 15 June 2023). **Special Eurobarometer 412:** Sport and physical activity, 2013. **Landing Page:** http://ec.europa.eu/public_opinion/archives/eb_special_419_400_en.htm#412 (accessed on 15 June 2023).

## Appendix A

**Table A1:** Life satisfaction among women of reproductive age (15–49 years) based on a European representative sample from Eurobarometer 2022, 2018 and 2013 (N = 18,489) classified by sociodemographic and sports variables.

			Life Satisfaction							Life Satisfaction			
Variables	Categories		Very Satisfied	Fairly Satisfied	Not Very Satisfied	Not at All Satisfied	Variables	Categories		Very Satisfied	Fairly Satisfied	Not Very Satisfied	Not at All Satisfied
Survey years ^#^ *p* < 0.001 **	2022	%	25.7	61.6	10.8	1.8	Marital status *p* < 0.001	(Re)Married	%	25.2	60.4	12.0	2.4
		N	1448	3475	611	104			N	2273	5451	1081	216
		CI	23.4–28.0	60.0–63.2	8.3–13.3	−0.8–4.4			CI	23.4–27.0	59.1–61.7	10.1–13.9	0.4–4.4
	2017	%	24.3	62.6	11.4	1.7		Single living with a partner	%	25.3	60.0	12.5	2.2
		N	1499	3861	702	108			N	813	1931	401	70
		CI	22.1–26.5	61.1–64.1	9.0–13.8	−0.7–4.1			CI	22.3–28.3	57.8–62.2	9.3–15.7	−1.2–5.6
	2013	%	22.8	55.8	16.8	4.5		Single	%	24.3	59.8	13.1	2.9
		N	1526	3730	1122	301			N	1138	2805	614	134
		CI	20.7–24.9	54.2–57.4	14.6–19.0	2.2–6.8			CI	21.8–26.8	58.0–61.6	10.4–15.8	0.1–5.7
Regularity of sports *p* < 0.001 **	Regularly	%	32.8	53.3	11.9	2.0		Divorced or separated	%	13.1	56.1	24.3	6.5
		N	370	601	134	22			N	153	652	282	76
		CI	28.0–37.6	49.3–57.3	6.4–17.4	−3.9–7.9			CI	7.8–18.4	52.3–59.9	19.3–29.3	1.0–12.0
	With some regularity	%	32.0	59.0	7.8	1.1		Widow	%	10.3	55.5	26.5	7.7
		N	2126	3917	521	76			N	14	77	37	11
		CI	30.0–34.0	57.5–60.5	5.5–10.1	−1.2–3.4			CI	5.3–15.3	44.4–66.6	12.3–40.7	−8.1–23.5
	Seldom	%	22.0	64.0	12.2	1.8		Other	%	32.0	57.5	7.8	2.7
		N	880	2566	489	71			N	84	151	21	7
		CI	19.3–24.7	62.1–65.9	9.3–15.1	−1.3–4.9			CI	20.9–43.1	49.6–65.4	−3.7–19.3	−9.3–14.7
	Never	%	16.4	59.3	19.2	5.1	Household members *p* < 0.001	One	%	21.1	59.6	15.7	3.7
		N	1097	3982	1291	344			N	377	1067	281	67
		CI	14.2–18.6	57.8–60.8	17.1–21.3	2.8–7.4			CI	13.2–29.0	56.7–62.5	11.4–20.0	−0.8–8.2
Social status *p* < 0.001 **	Working class	%	15.8	55.0	23.3	5.9		Two	%	22.5	59.8	14.3	3.5
		N	820	2855	1218	314			N	873	2315	555	136
		CI	13.3–18.3	53.2–56.8	20.9–25.7	3.3–8.5			CI	19.6–25.4	57.8–61.8	11.4–17.2	0.4–6.6
	Lower middle class	%	23.7	62.0	12.4	1.9		Three	%	22.7	61.1	13.6	2.6
		N	1276	3339	672	104			N	1084	2917	654	127
		CI	21.4–26.0	60.4–63.6	9.9–14.9	−0.7–4.5			CI	20.7–24.7	59.3–62.9	11.0–16.2	−0.2–5.4
	Middle class	%	27.5	64.3	7.1	1.1		Four or more	%	26.6	59.4	11.7	2.3
		N	1831	4284	474	74			N	2140	4768	945	184
		CI	25.5–29.5	62.9–65.7	4.8–9.4	−1.3–3.5			CI	24.6–28.6	58.0–60.8	9.7–13.7	0.1–4.5
	Upper middle class	%	47.0	48.3	3.8	0.8	Residence *p* = 0.581	Rural area or village	%	24.6	59.5	13.1	2.8
		N	472	485	39	8			N	1378	3340	733	159
		CI	42.5–51.5	43.9–52.7	−2.2–9.8	−5.4–7.0			CI	22.6–26.6	57.8–61.2	10.7–15.5	0.2–5.4
	Higher class	%	33.5	46.3	14.5	5.8		Small/middle town	%	24.0	60.3	13.2	2.5
		N	74	103	32	13			N	1724	4329	948	181
		CI	22.7–44.3	36.7–55.9	2.3–26.7	−6.9–18.5			CI	22.0–26.0	58.8–61.8	11.0–15.4	0.2–4.8
Difficulty paying bills *p* < 0.001 **	Most of the time	%	8.5	43.4	34.9	13.2		Large town	%	24.1	59.6	13.3	3.0
		N	186	946	760	286			N	1371	3398	755	174
		CI	4.5–12.5	40.2–46.6	31.5–38.3	9.3–17.1			CI	22.6–25.6	58.0–61.2	10.9–15.7	0.5–5.5
	From time to time	%	13.6	64.9	18.9	2.6	Terminal education age (years) *p* < 0.001	0	%	15.2	67.4	17.4	0.0
		N	810	3856	1122	156			N	7	29	8	0
		CI	11.2–16.0	63.4–66.4	16.6–21.2	0.1–5.1			CI	−11.4–41.8	50.3–84.5	−8.9–43.7	0.0–0.0
	Almost never/never	%	33.5	60.5	5.3	0.7		15- or less	%	11.7	52.6	27.6	8.1
		N	3478	6265	553	72			N	134	592	309	88
		CI	31.9–35.1	59.3–61.7	3.4–7.2	−1.2–2.6			CI	6.3–17.1	48.6–56.6	22.6–32.6	2.4–13.8
Employment *p* < 0.001 **	Self-employed	%	27.5	59.4	10.3	2.8		16–19	%	17.7	62.3	16.5	3.5
		N	334	721	126	34			N	1471	5055	1332	280
		CI	22.7–32.3	55.8–63.0	5.0–15.6	−2.7–8.3			CI	15.7–19.7	61.0–63.6	14.5–18.5	1.3–5.7
	Managers	%	34.2	58.8	6.2	0.8		20+	%	29.9	59.4	9.0	1.7
		N	929	1596	168	21			N	2300	4462	674	126
		CI	31.1–37.3	56.4–61.2	2.6–9.8	−3.0–4.6			CI	28.0–31.8	58.0–60.8	6.8–11.2	−0.6–4.0
	Other white collars	%	21.1	67.1	10.2	1.7		Still studying	%	34.1	57.7	7.0	1.2
		N	777	2475	375	62			N	561	928	112	19
		CI	18.2–24.0	65.2–69.0	7.1–13.3	−1.5–4.9			CI	30.2–38.0	54.5–60.9	2.3–11.7	−3.7–6.1
	Manual workers	%	19.5	62.2	15.6	2.6	Age categories *p* < 0.001	15–24	%	30.2	57.4	10.7	1.8
		N	913	2911	732	123			N	1008	1917	357	59
		CI	16.9–22.1	60.4–64.0	13.0–18.2	−0.2–5.4			CI	27.4–33.0	55.2–59.6	7.5–13.9	−1.6–5.2
	House persons	%	24.5	56.0	16.1	3.4		25–34	%	25.5	60.1	11.9	2.5
		N	461	1054	304	64			N	1361	3201	634	132
		CI	20.6–28.4	53.0–59.0	12.0–20.2	−1.0–7.8			CI	23.2–27.8	58.4–61.8	9.4–14.4	−0.2–5.2
	Unemployed	%	13.3	49.3	27.9	9.4		35–44	%	22.2	60.7	14.2	2.8
		N	225	831	470	159			N	1427	3900	915	178
		CI	8.9–17.7	45.9–52.7	23.8–32.0	4.9–13.9			CI	20.0–24.4	59.2–62.2	11.9–16.5	0.4–5.2
	Retired	%	11.8	50.3	28.8	9.2		45–49	%	19.9	60.2	15.6	4.2
		N	32	137	78	25			N	677	2048	530	144
		CI	0.6–23.0	41.9–58.7	18.8–38.8	−2.1–20.5			CI	16.9–22.9	58.1–62.3	12.5–18.7	0.9–7.5
	Students	%	34.1	57.0	7.7	1.1	Total		%	24.2	59.9	13.2	2.8
		N	803	1342	182	26			N	4474	11066	2435	513
		CI	30.8–37.4	54.4–59.6	3.8–11.6	−2.9–5.1			CI	22.9–25.5	59.0–60.8	11.86–14.5	1.4–4.2

CI: 95% confidence interval. ^#^ Pearson χ^2^, ** *p* ≤ 0.01.

## Appendix B

**Table A2:** Pre-test on the association of sport and sociodemographic variables with life satisfaction among women of reproductive age in a European representative sample—aggregated data from Eurobarometer 2022, 2018 and 2013 (N = 18,489), derived from multivariate logistic regression analysis (stepwise forward selection procedure).

		B	S.E.	Wald	df	Sig.	Exp(B)
Variables	Sport settings	0.124	0.024	26.081	1	<0.001 **	1.132
	Regularity of sports	−0.214	0.021	104.808	1	<0.001 **	0.807
	Social status	0.205	0.023	80.986	1	<0.001 **	1.227
	Difficulty paying bills	0.829	0.035	577.491	1	<0.001 **	2.292
	Household members	0.091	0.020	20.971	1	<0.001 **	1.095
	Residence	−0.088	0.024	13.728	1	<0.001 **	0.916
	Terminal education age	0.289	0.030	93.586	1	<0.001 **	1.336
	Marital status	−0.150	0.020	53.675	1	<0.001 **	0.861
	Survey years	0.094	0.025	13.756	1	<0.001 **	1.098
	Occupation	−0.049	0.011	19.866	1	<0.001 **	0.952
	Age categories	−0.173	0.022	61.025	1	<0.001 **	0.841
	Constant	−3.432	0.203	285.977	1	<0.001 **	0.032

** *p* < 0.01.
